# Description of *Deladenus hyrcanus* sp. n. (Tylenchomorpha, Neotylenchidae) from Iran

**DOI:** 10.2478/jofnem-2025-0037

**Published:** 2025-09-19

**Authors:** Mohammad Esmaeil Agh Atabai, Farzad Aliramaji, Ebrahim Shokoohi

**Affiliations:** Department of Plant Protection, Faculty of Plant Production, Gorgan University of Agricultural Sciences and Natural Resources, Gorgan, Iran; Department of Biochemistry, Microbiology and Biotechnology, University of Limpopo, Private Bag X1106, Sovenga 0727, South Africa

**Keywords:** *Deladenus gilanica*, fungivorous, morphology, nematodes, rDNA, taxonomy

## Abstract

During a survey on the members of the family Neotylenchidae in northern Iran, a population of *Deladenus* was recovered, which, based on the morphology and molecular characters, identified as a new species. *Deladenus hyrcanus* sp. n., was isolated from dead wood of *Acer velutinum* in Balaband forests, Mazandaran Province. Morphologically, the new species is characterized by the moderate body length of the mycetophagous females (718–806 μm) with c = 10.2–12.6, c′ = 3.5–4.4 and V = 86–87, stylet length 7.5–10 μm with three small knobs inclined backward, the lateral field with 7–8 incisures, position of excretory pore at level of hemizonid or slightly posterior to it, lacking a post-uterine sac, elongate-conoid tail (58–72 μm), with rounded to pointed terminus and males not observed. According to similarities in general morphology, *D. hyrcanus* sp. n., resembles *D. gilanica* and *D. zyzyphus*; however, the new species is distinguished from them in body length, lateral field incisures, and tail length. It was furthermore compared with *D. aridus*, *D. bonabensis* and *D. brevis*, by similarities in the position of the excretory pore and hemizonid, posterior to the nerve ring, lack of a median chamber, and a conoid tail narrowing to a rounded to pointed terminus. Molecular phylogenetic analysis based on D2-D3 expansion domain of 28S rDNA placed the new species close to *D. gilanica* in a clade with 1.00 posterior probability. The measurements, line illustration, and LM photographs are provided for the new species.

Neotylenchidae Thorne, 1941, is a family of nematodes historically recognized with harboring some tentative biological control candidates in agricultural pest management (Stock and Goodrich-Blair, 2012). Some members of this family exhibit a unique life cycle that includes both parasitic and free-living stages. In their parasitic phase, they parasitize insects or plants. Conversely, in their free-living stage, they inhabit various environments, e.g., soil and organic matter and feed on fungi (Siddiqi, 2000). Among the Neotylenchidae, at least one species of *Deladenus* Thorne, 1941, e.g., *D. siricidicola* Bedding, 1968, has been studied and used for controlling the pine-killing wood wasp *Sirex noctilio* (Hymenoptera: Siricidae) in Australia, South America, and South Africa (Hajek and Van Frankenhuyzen, 2017).

So far, eight species of *Deladenus* have been reported and described from Iran. Five species including *D. bonabensis* Amiri Bonab, Atighi and Pedram, 2023; *D. brevis* Heydari, Abolafia and Pedram, 2020; *D. gilanica* Jalalinasab, Esmaeili, Ye and Heydari, 2020; *D. hebetocaudatus* Rezaei, Pourjam, Atighi, Kanzaki, Giblin-Davis, and Pedram, 2024; *D. persicus* Miraeiz, Heydari and Golhasan, 2017; *D. tonekabonensis* Aliramaji, Taheri, and Shokoohi, 2024 are described; and two species including *D. apopkaetus* Chitambar, 1991 and *D. durus* (Cobb, 1922) Thorne, 1941 are reported from the country (Jahanshahi et al., 2014; Miraeiz et al., 2017; Heydari et al., 2020; Jalalinasab et al., 2020; Amiri Bonab et al., 2023; Aliramaji et al., 2024, Rezaei et al., 2024).

The present study was conducted in 2023 on the collected samples of deadwood from Balaband forests in Mazandaran Province, northern Iran and aims to 1) study the morphology of the newly recovered species of *Deladenus*, and 2) study its molecular phylogenetic relationships with other species using 28S rDNA D2-D3.

## Materials and Methods

### Nematode extraction and morphological observations

Wood and bark samples harboring wood-boring beetles were collected from the dead trunk of an *Acer velutinum* Boiss tree. Samples were chopped into small pieces less than 1 cm. The nematodes were extracted using the tray method (Whitehead and Hemming, 1965), fixed with hot 4% formaldehyde solution, and processed to anhydrous glycerin (De Grisse, 1969). Measurements, drawings, and light microphotographs were prepared using an Olympus BX51 light microscope equipped with a drawing tube and a digital camera.

### Molecular techniques and phylogenetic analyses

DNA was extracted from a single female. The nematode was crushed in TE buffer (10 mM Tris-Cl, 0.5 mM EDTA; pH 9.0, Qiagen) on a clean slide with a cover slip and the pressure of a plastic probe. The supernatant was extracted from the tube and stored at −20 °C. Primers for 28S rDNA (D2-D3 segment) amplification were forward primer D2Ab (5′-ACAAGTACCGTGAGGGAAAGT-3′) and reverse primer D3B (5′-TGCGAAGGAACCAGCTACTA-3′) (De Ley et al., 1999), with the following program: initial denaturation for 2 min at 94 °C, 35 cycles of denaturation for 30 s at 94° C; 55 °C annealing temperature for 45 s, and 3 min at 72 °C, and finally an extension step of 10 min at 72 °C followed by a temperature on hold at 4 °C (Aliramaji et al., 2019). The successfully amplified products were purified and sequenced directly for both strands using the same primers with an ABI 3730XL sequencer. The newly generated D2-D3 sequence of the new species (PV267324) was compared with those of other nematode species available in GenBank using the BLAST homology search program. The new sequence, together with the sequences retrieved from the GenBank database, were aligned using the Q-INSi algorithm of the online version of MAFFT version 7 (http://mafft.cbrc.jp/alignment/server/) (Katoh and Standley, 2013). The Gblocks program (version 0.91b) with all three less stringent parameters (http://phylogeny.lirmm.fr/phylo_cgi/one_task.cgi?task_type=gblocks) was used for post-editing of the alignment, i.e., to eliminate poorly aligned regions or divergent positions. The model of base substitution was selected using MrModeltest 2 (Nylander, 2004). The Akaike-supported model was used in 28S rDNA analysis. A general time-reversible model that included among-site rate heterogeneity and estimates of invariant sites (GTR + G + I) was used in the phylogeny. Bayesian analysis was performed using MrBayes v3.1.2 (Ronquist and Huelsenbeck, 2003), running the chains for 6 × 10^6^ generations. After discarding burn-in samples, the remaining samples were retained for further analyses. The Markov chain Monte Carlo (MCMC) method within a Bayesian framework was used to estimate the posterior probabilities of the phylogenetic trees (Larget and Simon, 1999) using the 50% majority rule. *Oscheius myriophilus* Poinar, 1986 (AY602176) and *Poikilolaimus piniperdae* Fuchs, 1930 (DQ059060) were used as outgroup taxa. The phylogenetic program output file was visualized using Dendroscope V.3.2.8 (Huson and Scornavacca, 2012), and the tree was digitally drawn in CorelDRAW v. 2017.

## Results

*Deladenus hyrcanus* sp. n.

LSID: urn:lsid:zoobank.org:pub:1F8069F8-C29C-4E89-A05B-61EC53860584.

Figures 1–3.

**Figure 1:**
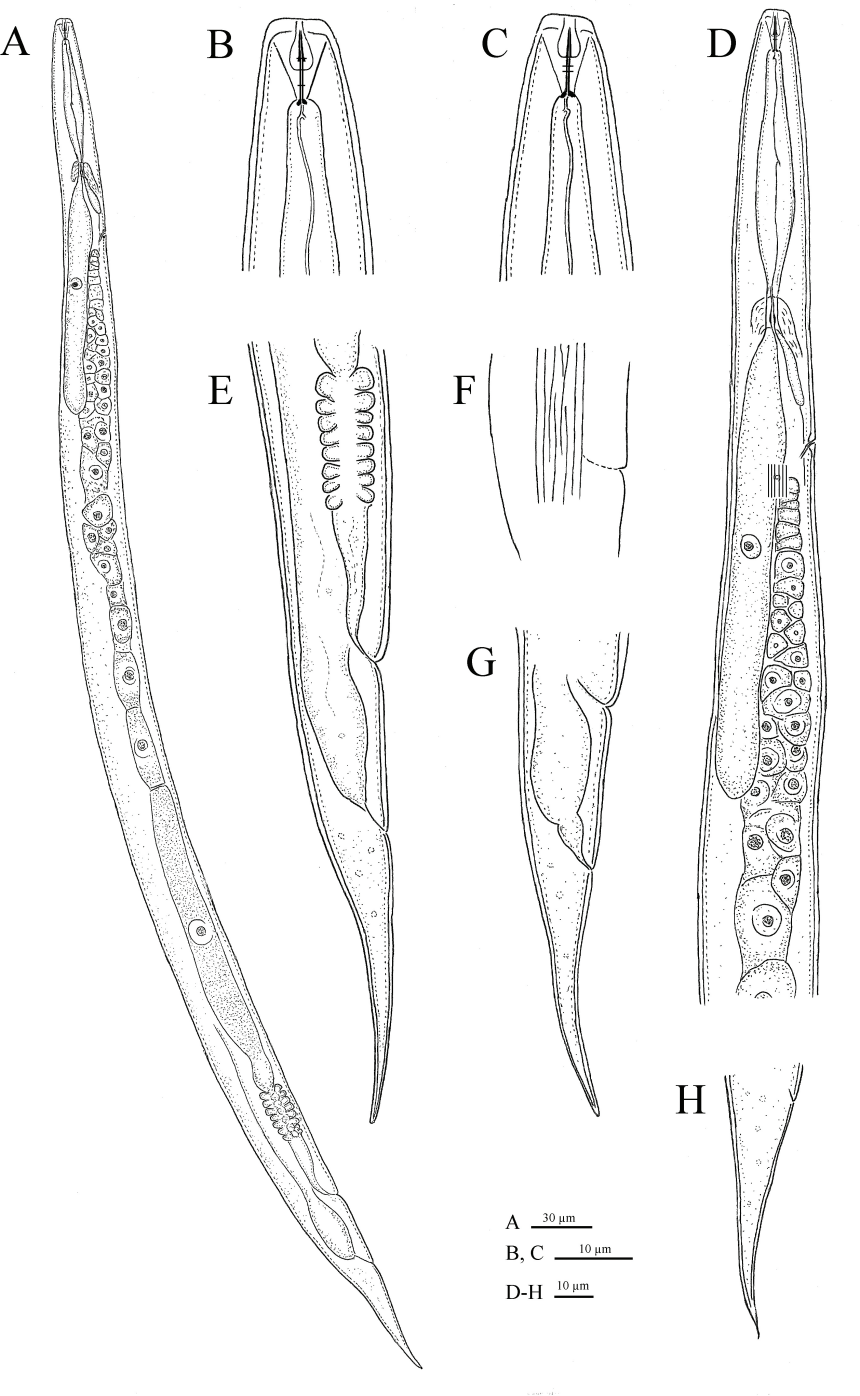
Mycetophagous female of *Deladenus hyrcanus* sp. n. A: Entire body; B, C: Anterior end; D: Pharyngeal region; E: Posterior body region and part of the reproductive system; F: Lateral lines; G, H: Posterior body region and tail.

**Figure 2:**
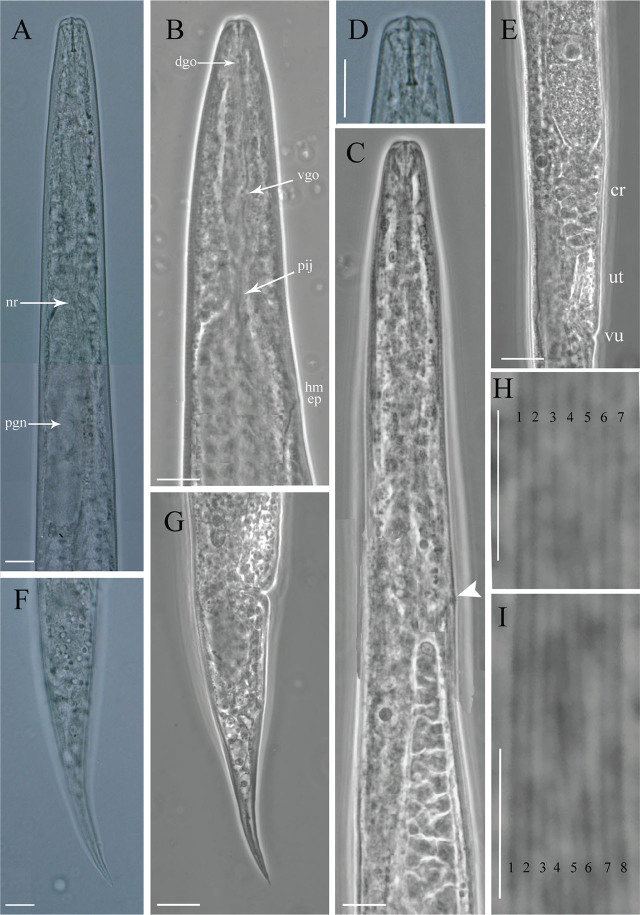
Mycetophagous female of *Deladenus hyrcanus* sp. n. A, C: Anterior region (pharynx); B: Anterior region (stylet, secretory-excretory pore, median bulb and part of pharynx); D: Anterior region (stylet); E: Posterior part of reproductive system containing crustaformeria, uterus, vulva; F, G: Posterior part of the body; H, I: Lateral lines. Abbreviation: cr = crustaformeria; dgo = dorsal gland orifice; ep: secretory-excretory pore; hm = hemizonid; nr: nerve ring; pij = pharyngo-intestinal junction; pgn: pharyngeal gland cell nucleus; sp: spermatheca; vgo = ventral glands orifice; ut = uterus; vu = vulva. all scale bars = 10 μm.

**Figure 3:**
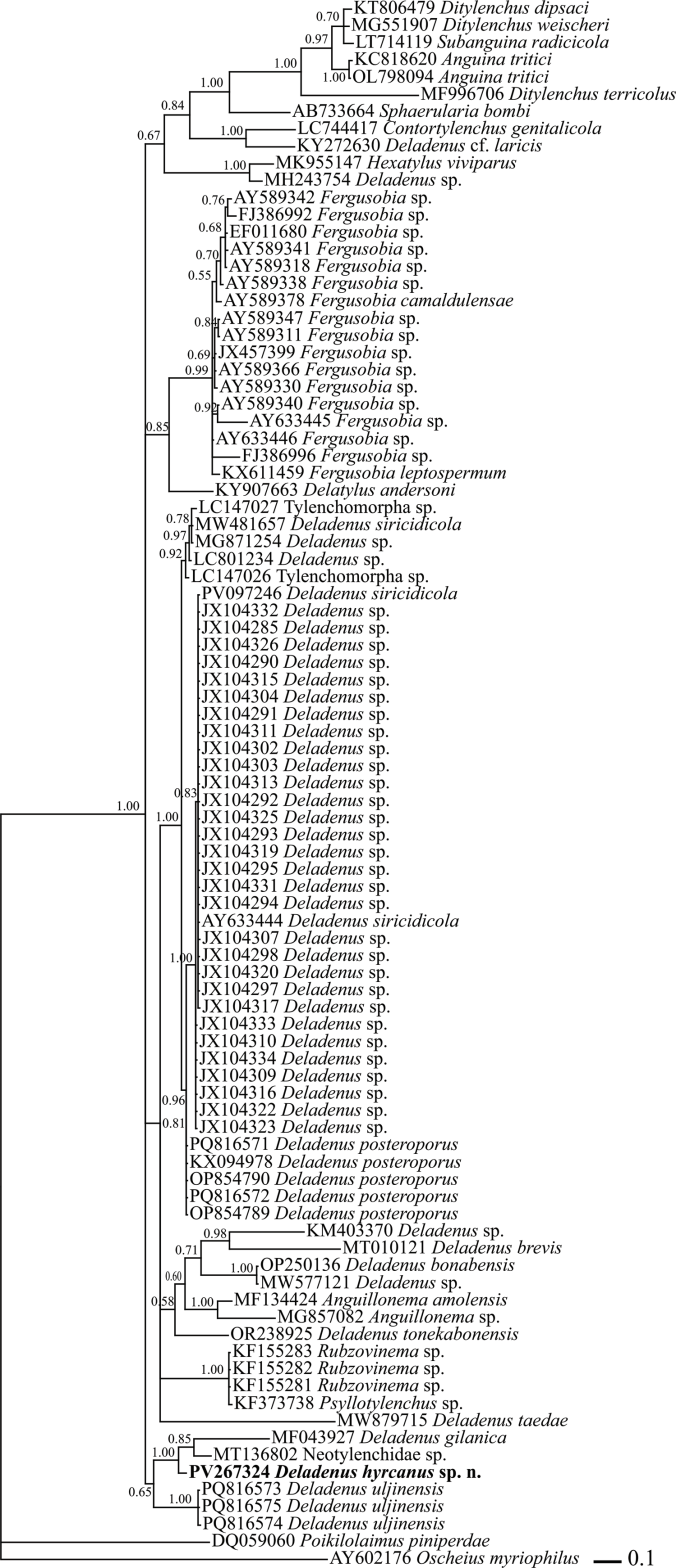
Bayesian 50% majority rule consensus tree of *Deladenus hyrcanus* sp. n. based on 28S rDNA D2-D3 segment sequences under GTR + I + G model. Bayesian posterior probability values more than 0.50 are given for appropriate clades. The new sequence is indicated in bold.

Measurements, See Table 1.

**Table 1:** Morphometrics of *Deladenus hyrcanus* sp. n. All measurements in μm and in the form: mean ± S.D. (range).

**Character**	**Female**	

	**Holotype**	**Paratypes**
n	1	7
L	759	752 ± 31.5 (718–806)
a	31.6	21.8 ± 2.6 (17.5–24.8)
b	9.0	9.4 ± 0.8 (8.5–10.5)
b′	3.8	4.2 ± 0.5 (3.8–5.1)
c	12.2	11.8 ± 0.8 (10.2–12.6)
c′	3.9	3.9 ± 0.3 (3.5–4.4)
V	86.3	86.3 ± 0.6 (86–87)
Lip region height	1.5	1.7 ± 0.2 (1.5–2.0)
Lip region width	7.5	7.7 ± 0.4 (7–8)
Stylet length	9	8.7 ± 0.8 (7.5–10.0)
Stylet conus	3	3.2 ± 0.4 (2.5–3.5)
Excretory pore from anterior end	112.5	110 ± 3.8 (106–115)
Hemizonid from anterior end	111	108.5 ± 3.1 (106–113)
Pharynx	84	80.0 ± 4.9 (71–86)
Nerve ring from anterior end	83	77.7 ± 3.5 (73–83)
Pharynx overlapping	114	98.0 ± 14.1 (72–115)
Maximum body diam.	24	34.9 ± 4.9 (30–44)
Vulval body diam. (VBD)	23	24.9 ± 3.5 (21–31)
Vulva–body end	104	102.9 ± 3.5 (97–111)
Vulva to anus	42	38.8 ± 5.4 (28.5–46.0)
Anal body diam.	16	16.4 ± 1.2 (15.0–18.5)
Tail length	62	64.0 ± 4.3 (58–72)

### Description

#### Mycetophagous female

Body almost straight after heat relaxation, gradually tapered toward both ends, more so toward posterior end by having an elongate conoid tail, not swollen at vulva. Cuticle with fine transverse striae, annulations less than one μm at mid body. Lateral field with seven to eight incisures at mid-body, sometimes oblique discontinuous middle incisures were observed. Deirids at middle of lateral field, 4–6 μm posterior to excretory pore. Cephalic region low (height/width = 0.2), flattened, with rounded sides, and continuous with body contour. Stylet slender, with distinct and posteriorly directed small basal knobs, its conus occupies *ca* 33 to 41% of its total length. Guiding apparatus complex (Fig. 2D). Orifice of dorsal pharyngeal gland 1.5–2.0 μm posterior to stylet knobs. Corpus fusiform, non-muscular, valveless, without median chamber. Subventral gland orifice clear (in phase contrast mode or fresh material), located at two-thirds of pharynx length posterior to stylet base (Fig. 2B), dorsal pharyngeal gland long, overlapping intestine dorsally, with only one nucleus visible, subventral glands shorter. The pharyngo-intestinal junction at nerve ring level, in some specimens, slightly posterior to it, simple, no specialized structure. Secretory excretory pore posterior to nerve ring, at the level with hemizonid or slightly posterior to it. Nerve ring usually at the same level with pharyngo-intestinal junction. Reproductive system monodelphic-prodelphic, ovary outstretched with one to two and three rows of oocytes in the germinal zone and a single row in growth zone, oviduct narrow, tube-like and long, spermatheca invisible, crustaformeria with eight cells in each of the four rows, uterus a thick-walled tube, vagina oblique anteriorly with moderately sclerotized walls, vulva as a wide transverse slit, without post-uterine sac (PUS). Rectum and anus distinct. Tail elongate conoid, 1.4–2.5 times the vulva-anus distance, narrowing to a rounded or, in some specimens, pointed tip.

*Mycetophagous male*: Not found.
*Infective female*: Not found.
*Parasitic female*: Not found.

#### Type host and locality

The new species was recovered from dead wood of an *Acer velutinum* tree in Balaband forests of Tonekabon city in Mazandaran Province, northern Iran (GPS coordinates: N: 36°45′32.312″; E: 50°41′4.648″). The first author collected the samples on 20 June 2023.

#### Etymology

The specific epithet is derived from the Hyrcanian forests where the new species of *Deladenus* was collected.

#### Type materials

The holotype female and three paratype females were deposited in the nematode collection of the Plant Protection Department, College of Plant Production, Gorgan University of Agricultural Sciences and Natural Resources, Iran. Two paratype females were deposited at the Nematology collection of the Aquaculture Research Unit of the University of Limpopo, South Africa and two paratype females were deposited in the WaNeCo nematode collection, of the Wageningen University, The Netherlands (http://www.waneco.eu).

#### Diagnosis and relationships

The new species is characterized by moderate body length of mycetophagous females (718–806 μm), their c = 10.2–12.6, c′ = 3.5–4.4 and V = 86–87, short stylet (7.5–10 μm) with three knobs inclined backward, corpus without median chamber, 7–8 incisures in lateral field, excretory pore posterior to nerve ring, at the level of hemizonid or slightly posterior it, lacking a PUS and elongate conoid tail (58–72 μm) narrowing to a rounded to pointed terminus.

By similarities in general morphology, position of excretory pore and hemizonid posterior to nerve ring, lack of median chamber and conoid tail narrowing to a rounded to pointed terminus, the new species resembles *D. aridus* Andrássy, 1957; *D. bonabensis*; *D. brevis*; *D. gilanica* and *D. zyzyphus* Bajaj, 2015.

Compared with *D. aridus*, the new species differs in vulva position (V = 86–87 vs 91.9), number of lateral field incisures (7–8 vs 4), and longer tail (c = 10.2–12.6 vs 19.8).

It differs from *D. bonabensis* by shorter body (718–806 vs 1051–1185 μm), number of lateral field incisures (7–8 vs 6), longer tail (58–72 vs 36–46 μm), and vulva position (86–87 vs 93.5–95).

Compared with *D. brevis*, the new species differs in longer body (718–806 vs 367–454 μm), longer stylet (7.5–10 vs 6–7 μm), number of lateral fields incisures (7–8 vs 6), position of excretory pore (posterior vs at the level with nerve ring), longer tail (58–72 vs 20–30 μm) and vulva without lateral flaps vs existence of small lateral flaps.

It differs from *D. gilanica* by longer body (718–806 vs 314–422 μm), number of lateral field incisures (7–8 vs 8), longer tail (58–72 vs 27–32 μm) and vulva to anus distance (28.5–46 vs 15–26 μm).

The new species differs from *D. zyzyphus* by shorter body (718–806 vs 790–1110 μm), number of lateral field incisures (7–8 vs 4), stouter body (a = 17.5–24.8 vs 45–51), lower c′ ratio (3.5–4.4 vs 6.1–7.0), and shorter tail (58–72 vs 69–90 μm).

### Molecular phylogenetic status

Sequencing of the D2-D3 expansion region of the new species was performed using both reverse and forward promers, and the final 785 bp long sequence was reconstructed by aligning both reads. The BLAST search of this new sequence showed it has 84.56% identity with the corresponding sequence of the Korean population of *Deladenus posteroporus* Yu, Gu, Ye, Li and He 2017 (PQ816571), 84.46% identity with the Chinese population of *Deladenus posteroporus* (KX094978), 84.21% identity with *Deladenus* sp. (LC801234), 86.21% identity with an unidentified neotylenchid species (MT136802), 84.14% identity with Tylenchomorpha sp. (LC147027) and 83% identity with some Siricidicola group of *Deladenus* (JX104293, JX104313, JX104311, JX104326, MW481657). In the reconstructed 28S rDNA phylogenetic tree (Fig. 3), *Deladenus* species were distributed in several clades. However, the newly generated sequence of *D. hyrcanus* sp. n. formed a maximally supported clade with a clade including sequences *D. gilanica* (MF043927) and Neotylenchidae sp. (MT136802).

## Discussion

In the present study, a new species of *Deladenus* has described from Iran. This finding suggests that the genus *Deladenus* may be more diverse in Iran than currently recognized. The new species has been characterized based on its free-living phase. Formerly, several species of *Deladenus* have historically been described in this manner (Aliramaji et al., 2024). As known, *Deladenus* exhibits two distinct life cycles: a free-living mycetophagous phase and an insect-parasitic phase (Siddiqi, 2000). The earlier species described from Iran were also based on their free-living stage; notably, *D. gilanica* was described using both its free-living and infective phases (Jalalinasab et al., 2020). The present phylogenetic analysis based on the D2–D3 expansion domains of 28S rDNA revealed that the currently sequenced *Deladenus* species are distributed across several distinct clades, consistent with previous studies (e.g., Kanzaki et al., 2016; Amiri Bonab et al., 2023; Aliramaji et al., 2024).
